# Maternal Serum Alpha-Fetoprotein as a Predictor of Persistent Low-Lying Placenta and Placenta Accreta Spectrum: A Prospective Observational Study

**DOI:** 10.7759/cureus.113514

**Published:** 2026-07-28

**Authors:** Nupur Prakash, Ankit Karan

**Affiliations:** 1 Department of Obstetrics and Gynecology, Anugrah Narayan Magadh Medical College, Gaya, IND; 2 Department of Internal Medicine, Indira Gandhi Institute of Medical Sciences, Patna, IND

**Keywords:** abnormal placentation, antepartum hemorrhage, cesarean delivery, low lying placenta, maternal serum alpha fetoprotein, obstetric outcomes, placenta accreta spectrum, placenta previa, prenatal screening, risk stratification

## Abstract

Introduction

Placenta previa and low-lying placenta are major contributors to antepartum hemorrhage and are strongly associated with placenta accreta spectrum, a leading cause of severe maternal morbidity. Early identification of pregnancies at risk remains clinically important. Maternal serum alpha-fetoprotein (MS-AFP), routinely measured during antenatal screening, has been proposed as a biomarker of abnormal placentation. This study evaluated the association between maternal serum alpha-fetoprotein levels and persistence of low-lying placenta, as well as its predictive value for placenta accreta spectrum.

Methods

This observational study was conducted in the Department of Obstetrics and Gynaecology, Patna Medical College and Hospital, Patna, Bihar, India. Sampling was performed using a convenience sampling approach. Consecutive eligible pregnant women attending the Department of Obstetrics and Gynaecology between November 2019 and October 2021 who fulfilled the inclusion criteria were recruited until a total of 100 participants was achieved. One hundred pregnant women with ultrasonographically confirmed low-lying placenta between 15 and 20 weeks' gestation were enrolled. Maternal serum alpha-fetoprotein levels were measured and expressed as multiples of the median. Participants were followed until delivery to assess placental migration, occurrence of placenta accreta spectrum, and obstetric outcomes. Statistical analysis was performed using appropriate comparative tests, with statistical significance defined as a p-value less than 0.05.

Results

Among the 100 enrolled women, low-lying placenta persisted in 45 (45.0%), whereas placental migration occurred in 55 (55.0%). Subsequent analyses demonstrated that persistence was significantly associated with prior cesarean delivery and multiparity. Placenta accreta spectrum occurred in 13 (13.0%) women, all of whom had persistent low-lying placenta. Elevated maternal serum alpha-fetoprotein levels were present in 88.9% of persistent cases and in all cases of placenta accreta spectrum. MS-AFP values greater than 1.1 MoM were associated with persistence, while MS-AFP values exceeding 1.7 MoM (multiples of the median) were strongly related with the need for cesarean hysterectomy.

Conclusion

MS-AFP may serve as a useful adjunctive marker for identifying pregnancies at an increased risk of persistent low-lying placenta and placenta accreta spectrum. Larger prospective studies are needed to validate these findings. These findings suggest that MS-AFP may complement ultrasonographic assessment; however, further prospective studies are required to evaluate its additional diagnostic value.

## Introduction

Placenta previa refers to placental implantation in the lower uterine segment partially or completely covering the internal cervical os [1]. According to the National Institutes of Health (NIH) Fetal Imaging Workshop, a low-lying placenta (LLP) is defined as placental implantation within 2 cm of the internal os without covering it [1]. Both conditions are clinically significant because of their strong association with antepartum hemorrhage and adverse maternal and perinatal outcomes [2].

Placental development involves differentiation of trophoblasts into cytotrophoblasts and syncytiotrophoblasts, forming chorionic villi and the intervillous space. Under normal circumstances, placental separation occurs during the third stage of labor. In placenta previa, however, premature separation may occur during cervical effacement and dilatation, resulting in unpredictable hemorrhage [3]. Although the incidence of placenta previa is approximately 12% in the second trimester, it decreases to nearly 0.3% at term due to placental “migration,” a process attributed to preferential growth toward better-vascularized uterine regions and regression of poorly perfused placental tissue (trophotropism) [1,3,4]. Migration is less likely in posterior placentation, thick placental margins, and in women with previous cesarean delivery, thereby increasing the likelihood of persistence and related complications [4].

Placenta accreta spectrum (PAS) encompasses abnormal placental adherence to the myometrium, including placenta accreta, increta, and percreta. The International Federation of Gynecology and Obstetrics (FIGO) has proposed a standardized clinical classification for placenta accreta spectrum to improve consistency in diagnosis and reporting [5]. PAS is a major cause of severe obstetric complications, including massive hemorrhage, blood transfusion, peripartum hysterectomy, intensive care admission, and maternal mortality.

The increasing prevalence of PAS parallels rising cesarean delivery rates. Uterine scarring disrupts normal decidualization and the Nitabuch layer, facilitating abnormal trophoblastic invasion [6]. The coexistence of placenta previa further amplifies this risk. Additional contributors include advanced maternal age, multiparity, prior uterine curettage, smoking, and assisted reproductive techniques. The risk of PAS increases from approximately 3.3% in women with placenta previa and one prior cesarean delivery to nearly 40% in those with three or more cesarean sections [4,6].

Maternal serum alpha-fetoprotein (MS-AFP), a glycoprotein synthesized by the fetal yolk sac, liver, and gastrointestinal tract, is routinely used in screening for neural tube defects and chromosomal abnormalities. Increasing evidence suggests that unexplained elevations in MS-AFP are also associated with adverse pregnancy outcomes, including preeclampsia, placental abruption, preterm birth, and PAS [7]. Kupferminc et al. first demonstrated a significant association between elevated MS-AFP and abnormal placental adherence [8], with subsequent studies confirming this relationship, particularly in cases of placenta previa or LLP [9,10].

The proposed mechanism involves disruption of the maternal-fetal interface secondary to defective decidualization and absence of the Nitabuch layer, permitting excessive trophoblastic invasion and increased transplacental leakage of alpha-fetoprotein. Vascular injury and placental ischemia may further contribute to elevated MS-AFP levels [11]. Thus, the magnitude of MS-AFP elevation may reflect the severity of placental invasion.

Although ultrasonography remains the primary diagnostic modality for PAS, its accuracy is operator-dependent and may be limited in resource-constrained settings. Magnetic resonance imaging may provide additional diagnostic value but is not universally accessible. Therefore, incorporation of MS-AFP, an inexpensive and widely available biomarker, may enhance early risk stratification in women with low-lying placenta and facilitate timely referral, multidisciplinary planning, and optimized maternal and neonatal outcomes.

Given the established association between low-lying placenta, PAS, and elevated MS-AFP, this study aimed to evaluate the association between MS-AFP levels and persistent low-lying placenta and PAS, while also assessing maternal and obstetric factors associated with placental persistence and identifying clinically relevant MS-AFP cut-off values for predicting PAS and the need for cesarean hysterectomy.

## Materials and methods

Study design and setting

This prospective observational cohort study was conducted in the Department of Obstetrics and Gynaecology, Patna Medical College and Hospital, Patna, Bihar, India, between November 2019 and October 2021. Ethical approval was obtained from the Institutional Ethics Committee of Patna Medical College (Approval No. MF/855; dated September 18, 2020). Written informed consent was obtained from all participants prior to enrolment.

Study population

Pregnant women between 15 and 20 weeks of gestation with ultrasonographically diagnosed low-lying placenta attending the outpatient department or emergency services were screened for eligibility. Low-lying placenta was defined as placental implantation within 20 mm of the internal cervical os.

Women with vaginal bleeding due to causes other than abnormal placental implantation, including spontaneous abortion, placental abruption, molar pregnancy, cervical or vaginal pathology, cervical polyps, or retained foreign bodies, were excluded. Additional exclusion criteria included multiple pregnancy, major fetal structural anomalies, intrauterine fetal demise, hypertensive disorders of pregnancy, and gestational diabetes mellitus.

Sample size and data collection

A total of 100 consecutive eligible women fulfilling the inclusion criteria were enrolled during the study period. Participants were recruited consecutively until the predetermined sample size specified in the study protocol was achieved.

At enrolment, demographic characteristics, obstetric history, parity, previous cesarean delivery, history of abortion, and relevant clinical information were recorded using a structured proforma.

Three millilitres of maternal venous blood were collected between 15 and 20 weeks of gestation for estimation of maternal serum alpha-fetoprotein (MS-AFP) using a chemiluminescence immunoassay. The results were expressed as multiples of the median (MoM), calculated by dividing the measured MS-AFP concentration by the gestational age-specific median value provided by the reporting laboratory.

All participants underwent transabdominal ultrasonography for assessment of placental location. Transvaginal ultrasonography was performed whenever clinically indicated to improve visualization of the lower uterine segment. Placental location was reassessed during the third trimester to determine whether the low-lying placenta had migrated or persisted.

Outcome measures

The primary outcome was persistence of low-lying placenta until delivery. Secondary outcomes included placenta accreta spectrum (placenta accreta, increta, or percreta), preterm delivery, cesarean hysterectomy, and other maternal outcomes related to abnormal placentation. Placenta accreta spectrum was diagnosed based on intraoperative findings and confirmed by histopathological examination wherever hysterectomy specimens were available.

Clinical management****

Patients were managed according to institutional protocols based on gestational age, maternal haemodynamic status, severity of bleeding, fetal condition, and ultrasonographic findings. Conservative management was adopted whenever clinically feasible. The timing and mode of delivery were individualized according to maternal and fetal condition. Women with suspected placenta accreta spectrum were managed by a multidisciplinary team with preparation for possible cesarean hysterectomy.

Statistical analysis

Statistical analysis was performed using IBM SPSS Statistics for Windows, version 25.0 (IBM Corp., Armonk, NY, USA).

Continuous variables were expressed as mean±standard deviation (SD), while categorical variables were expressed as frequencies and percentages. Comparisons between groups were performed using Student's t-test for continuous variables and the chi-square test or Fisher's exact test for categorical variables, as appropriate.

A receiver operating characteristic (ROC) curve analysis was performed to evaluate the diagnostic performance of MS-AFP in predicting persistence of low-lying placenta. The optimal MS-AFP cut-off value was determined using the Youden index. Diagnostic performance was assessed by calculating the area under the receiver operating characteristic curve (AUC), sensitivity, specificity, positive predictive value (PPV), negative predictive value (NPV), positive likelihood ratio (LR+), and negative likelihood ratio (LR-). A two-sided p-value of <0.05 was considered statistically significant.

## Results

Study population

A total of 100 pregnant women with ultrasonographically diagnosed low-lying placenta between 15 and 20 weeks' gestation were enrolled in the study. Placental migration occurred in 55 (55.0%) women, whereas persistent placental abnormality at delivery was observed in 45 (45.0%).

The majority of participants were aged 21-30 years (75.0%), followed by >30 years (16.0%) and <20 years (9.0%). Maternal age was not significantly associated with persistent placental abnormality (p=0.344). Among the study population, 35 women were primigravidae and 65 were multigravidae. Persistence of placental abnormality was significantly more frequent among multigravidae than primigravidae (53.8% vs. 28.6%, p=0.015) (Table 1).

**Table 1:** Baseline Characteristics of the Study Population According to Placental Outcome Baseline characteristics of the study population according to placental outcome. Values are presented as number (%). Persistent placental abnormality includes persistent low-lying placenta, placenta previa, and placenta accreta spectrum diagnosed at delivery.

Characteristic	Placental migration (n=55)	Persistent placental abnormality (n=45)	Total (N=100)	*p* value
Maternal age (years)				0.344
<20	7 (77.8)	2 (22.2)	9 (9.0)	
21–30	40 (53.3)	35 (46.7)	75 (75.0)	
>30	8 (50.0)	8 (50.0)	16 (16.0)	
Gravidity				0.015
Primigravida	25 (71.4)	10 (28.6)	35 (35.0)	
Multigravida	30 (46.2)	35 (53.8)	65 (65.0)

A significant association was observed between previous cesarean delivery and persistent placental abnormality. Persistence occurred in 19 of 20 women (95.0%) with one previous cesarean delivery and in all women with two or more previous cesarean deliveries (p<0.001) (Table 2).

**Table 2:** Association Between Previous Cesarean Delivery and Persistent Placental Abnormality Association between previous cesarean delivery and placental outcome. Persistence of placental abnormality increased significantly with increasing number of previous cesarean deliveries.

Previous cesarean delivery	Placental migration n (%)	Persistent placental abnormality n (%)	Total n (%)
None	54 (74.0)	19 (26.0)	73 (100)
One	1 (5.0)	19 (95.0)	20 (100)
Two	0 (0.0)	6 (100.0)	6 (100)
Three	0 (0.0)	1 (100.0)	1 (100)
Total	55 (55.0)	45 (45.0)	100 (100)

Thirty women had a history of previous abortion. No significant association was observed between previous abortion and persistent placental abnormality (p=0.281).

Placental outcomes

Final placental diagnosis at delivery is summarized in Table 3. Placental migration occurred in 55 (55.0%) women, whereas persistent placental abnormality was identified in 45 (45.0%). Among women with persistent placental abnormality, 27 (60.0%) had persistent low-lying placenta, five (11.1%) had placenta previa, seven (15.6%) had placenta accreta, two (4.4%) had placenta increta, and four (8.9%) had placenta percreta.

**Table 3:** Final Placental Diagnosis at Delivery (N=100) Final placental diagnosis at delivery. Persistent placental abnormality was observed in 45 women (45.0%) and comprised persistent low-lying placenta, placenta previa, and placenta accreta spectrum disorders. Placental migration occurred in 55 women (55.0%). The final placental diagnosis was confirmed at delivery.

Final placental diagnosis	Number (n)	Percentage (%)
Placental migration (no persistent placental abnormality)	55	55
Persistent low-lying placenta	27	27
Placenta previa	5	5
Placenta accreta	7	7
Placenta increta	2	2
Placenta percreta	4	4
Total	100	100

**Figure 1:**
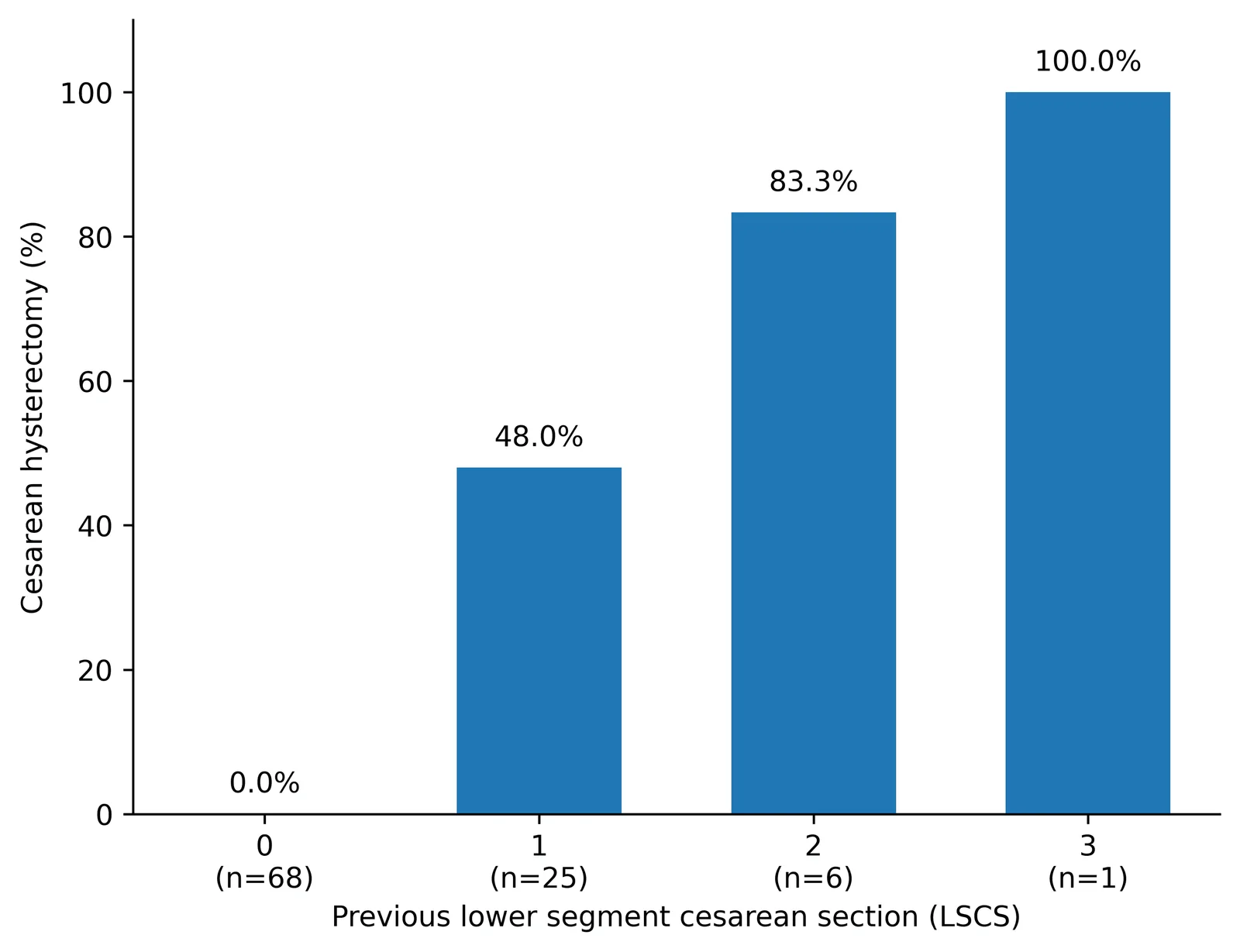
Frequency of Cesarean Hysterectomy According to the Number of Previous Cesarean Deliveries The proportion of women undergoing cesarean hysterectomy increased with increasing numbers of previous lower segment cesarean sections (LSCS).

Cesarean hysterectomy was performed in 18 women, all of whom belonged to the persistent placental abnormality group. The proportion of women requiring cesarean hysterectomy increased progressively with the number of previous cesarean deliveries, from 0% among women with no prior cesarean delivery to 100% among those with three previous cesarean deliveries (Figure 1).

Association between maternal serum alpha-fetoprotein and persistent placental abnormality

**Figure 2:**
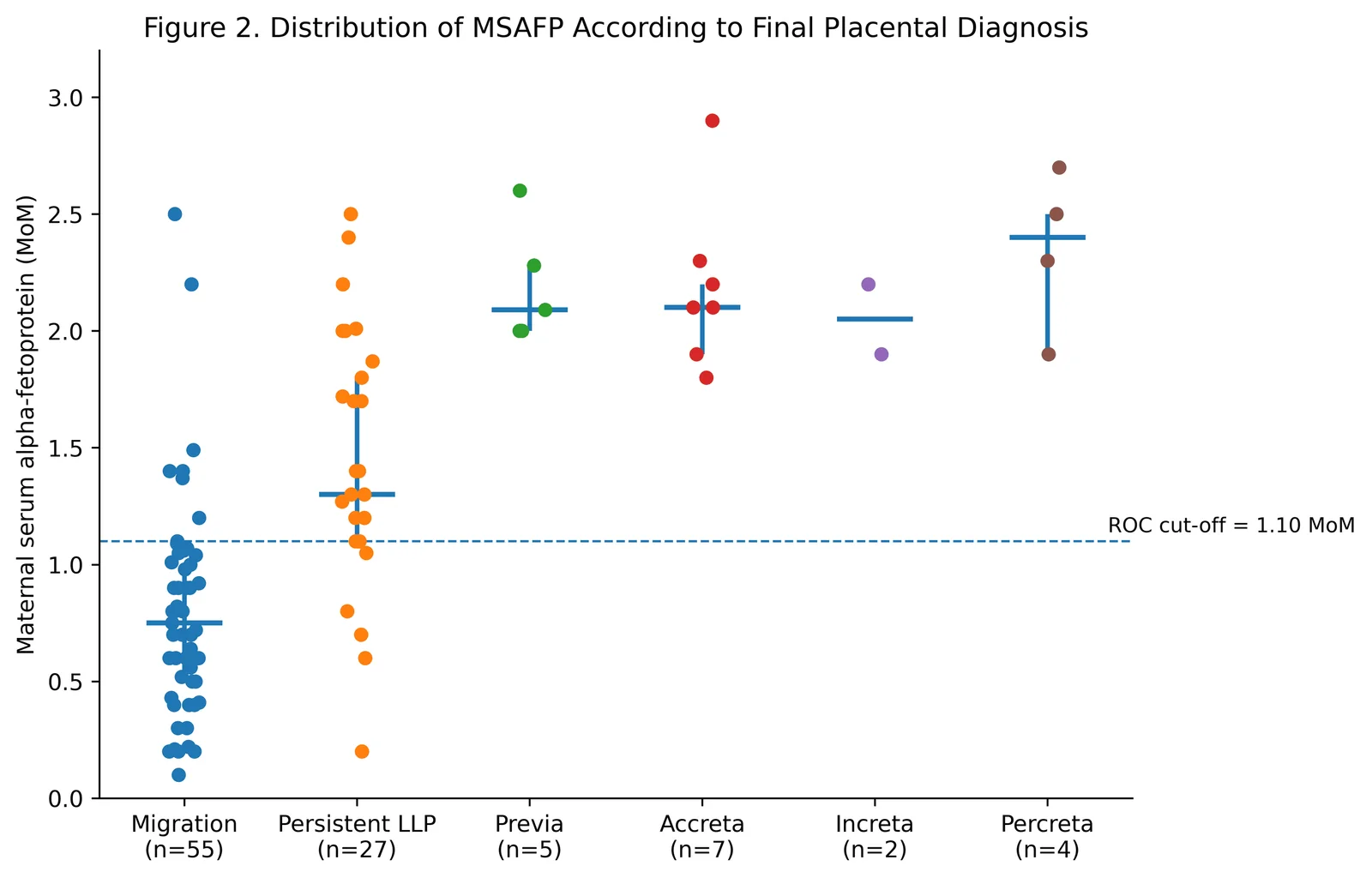
Distribution of Maternal Serum Alpha-Fetoprotein (MS-AFP) Levels According to Final Placental Diagnosis Individual maternal serum alpha-fetoprotein (MS-AFP) values are shown for each diagnostic group. The horizontal dashed line indicates the optimal ROC-derived cut-off value of 1.10 MoM. Horizontal bars represent the median and interquartile range.

Maternal serum alpha-fetoprotein (MS-AFP) levels demonstrated a significant association with persistent placental abnormality (p<0.001) (Table 4). Among women with normal MS-AFP levels (<1.10 MoM), only five of 52 (9.6%) developed persistent placental abnormality. In contrast, 40 of 48 (83.3%) women with elevated MS-AFP levels (≥1.10 MoM) had persistent placental abnormality. The distribution of individual MS-AFP values across the different final placental diagnoses is shown in Figure 2.

**Table 4:** Association Between Maternal Serum Alpha-Fetoprotein Levels and Persistent Placental Abnormality Association between maternal serum alpha-fetoprotein (MS-AFP) levels and placental outcome. Women with elevated MS-AFP levels (≥1.10 MoM) had a significantly higher frequency of persistent placental abnormality than women with normal MS-AFP levels.

Maternal serum alpha-fetoprotein (MoM)	Placental migration n (%)	Persistent placental abnormality n (%)	Total n (%)
Normal (<1.10)	47 (90.4)	5 (9.6)	52 (100)
Elevated (≥1.10)	8 (16.7)	40 (83.3)	48 (100)
Total	55 (55.0)	45 (45.0)	100 (100)

Diagnostic performance of maternal serum alpha-fetoprotein

**Figure 3:**
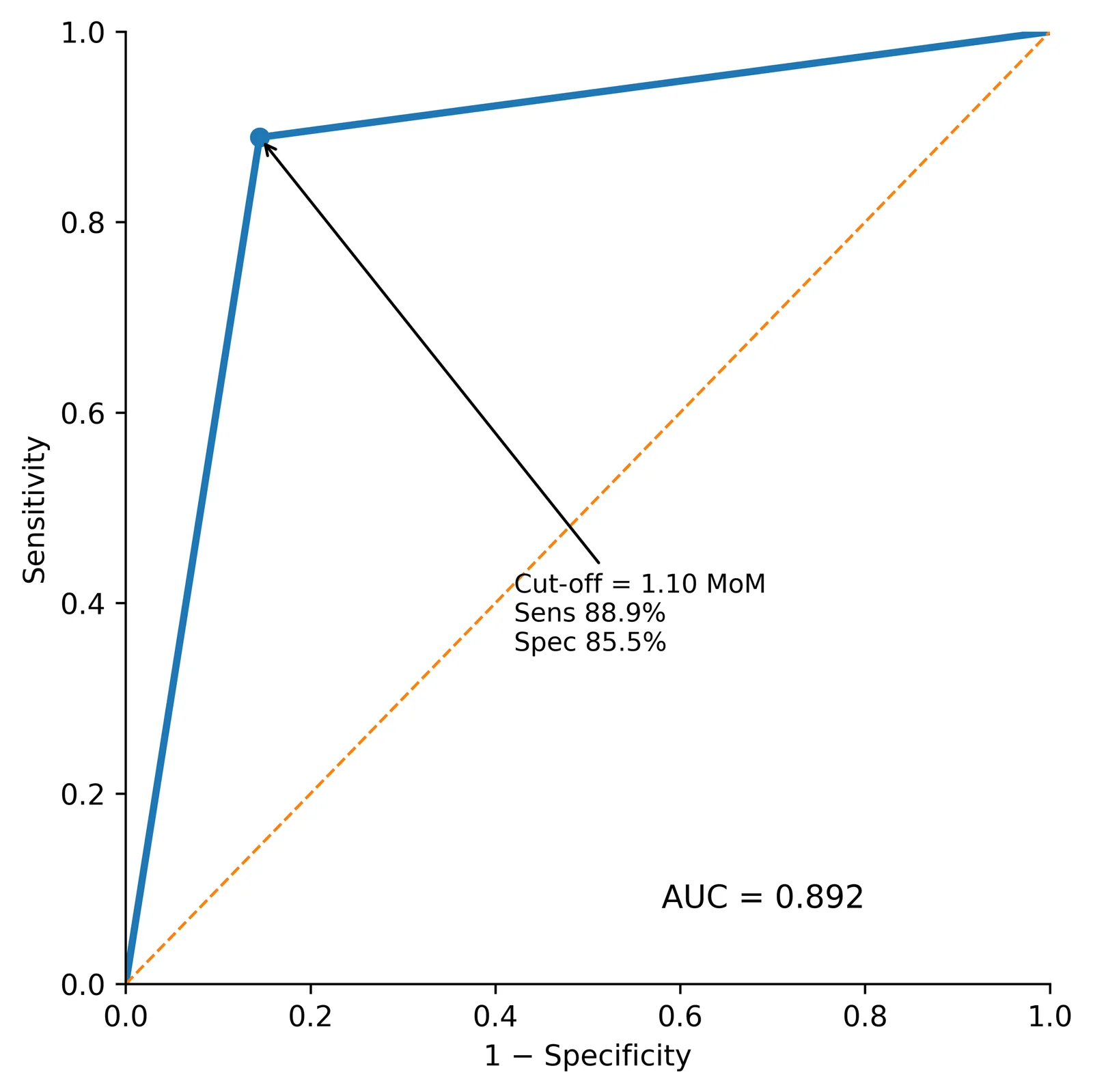
Receiver Operating Characteristic (ROC) Curve of Maternal Serum Alpha-Fetoprotein for Predicting Persistent Low-Lying Placenta Receiver operating characteristic (ROC) curve demonstrating the diagnostic performance of maternal serum alpha-fetoprotein (MS-AFP) in predicting persistent placental abnormality. The optimal cut-off value was 1.10 MoM (AUC=0.892), with a sensitivity of 88.9% and specificity of 85.5%.

Receiver operating characteristic (ROC) curve analysis demonstrated good diagnostic performance of MS-AFP for predicting persistent placental abnormality (Figure 3). The area under the ROC curve (AUC) was 0.892. The optimal cut-off value determined using the Youden index was 1.10 MoM, corresponding to a sensitivity of 88.9%, specificity of 85.5%, positive predictive value of 83.3%, negative predictive value of 90.4%, positive likelihood ratio of 6.11, and negative likelihood ratio of 0.13 (Table 5).

**Table 5:** Diagnostic Performance of Maternal Serum Alpha-Fetoprotein for Predicting Persistent Placental Abnormality Receiver operating characteristic (ROC) analysis of maternal serum alpha-fetoprotein for predicting persistent placental abnormality. The optimal threshold identified using the Youden index was 1.10 MoM. MS-AFP: Maternal serum alpha-fetoprotein; MoM: multiples of the median.

Parameter	Value
Optimal MS-AFP cut-off (MoM)	1.1
Area under ROC curve (AUC)	0.892
Sensitivity (%)	88.9
Specificity (%)	85.5
Positive predictive value (%)	83.3
Negative predictive value (%)	90.4
Positive likelihood ratio (LR+)	6.11
Negative likelihood ratio (LR−)	0.13

Effect size analysis

Effect size analysis identified elevated maternal serum alpha-fetoprotein (≥1.10 MoM) as the strongest predictor of persistent placental abnormality (Table 6). Previous cesarean delivery and multigravidity were also significantly associated with persistent placental abnormality, whereas previous abortion was not. 

**Table 6:** Effect Size Analysis of Clinical Predictors of Persistent Placental Abnormality Effect size analysis of clinical predictors of persistent placental abnormality. Odds ratios (ORs) and risk ratios (RRs) are presented with 95% confidence intervals (CIs). Elevated maternal serum alpha-fetoprotein (≥1.10 MoM) demonstrated the strongest association with persistent placental abnormality. LSCS: Lower segment cesarean sections; MoM: multiples of the median.

Variable	Odds Ratio (95% CI)	Risk Ratio (95% CI)	p value
Previous LSCS (≥1 vs none)	15.00 (5.01–44.87)	4.71 (2.08–10.66)	<0.001
Multigravidity vs primigravidity	6.74 (2.46–18.49)	2.01 (1.45–2.80)	<0.001
MS-AFP ≥1.10 MoM	47.00 (14.24–155.15)	5.42 (2.86–10.27)	<0.001
Previous abortion (≥1 vs none)	2.39 (1.00–5.73)	1.54 (0.95–2.47)	0.079

Table 7 summarizes the clinical interpretation of the MS-AFP cut-off values and the corresponding suggested clinical approach for each range.

**Table 7:** Clinical Interpretation of Maternal Serum Alpha-Fetoprotein (MS-AFP) Cut-off Values in Women with Low-Lying Placenta Abbreviations: MoM, Multiples of the median; PAS, placenta accreta spectrum; ROC, receiver operating characteristic; AUC, area under the curve. Suggested clinical approaches are based on the findings of the present study and should be interpreted as adjuncts to established obstetric management rather than standalone recommendations.

MS-AFP (MoM)	Interpretation	Observed Outcome in the Present Study	Suggested Clinical Approach*
<1.10	Lower likelihood of persistent placental abnormality	Most women experienced placental migration	Routine antenatal follow-up with repeat ultrasonography to confirm placental migration.
≥1.10	Increased likelihood of persistent placental abnormality	Associated with persistent low-lying placenta (ROC-derived optimal cut-off; AUC = 0.892, sensitivity 88.9%, specificity 85.5%)	Repeat targeted ultrasonography, closer surveillance, and assessment for placenta accreta spectrum (PAS) in women with additional risk factors (e.g., previous cesarean delivery).
≥1.70	Higher likelihood of PAS	Associated with placenta accreta spectrum and an increased likelihood of cesarean hysterectomy	Referral to a tertiary care center, detailed PAS evaluation by an experienced fetal medicine team, and multidisciplinary delivery planning.

## Discussion

The present study evaluated the association between MS-AFP levels and the persistence of low-lying placenta diagnosed during the second trimester. Persistent placental abnormality was observed in 45% of women, whereas placental migration occurred in 55%. Persistent placental abnormality was significantly associated with previous cesarean delivery, multigravidity, elevated MS-AFP levels, placenta accreta spectrum (PAS), and cesarean hysterectomy. Furthermore, receiver operating characteristic (ROC) analysis demonstrated good diagnostic performance of MS-AFP for predicting persistent placental abnormality, suggesting that it may be a useful adjunctive biomarker for antenatal risk stratification.

The placental migration rate observed in the present study is consistent with previous reports demonstrating that most low-lying placentas diagnosed during the second trimester migrate away from the internal cervical os before delivery. Progressive development of the lower uterine segment and differential placental growth during pregnancy contribute to this phenomenon. However, persistence of a low-lying placenta is associated with an increased risk of placenta previa, PAS, antepartum hemorrhage, and operative delivery, emphasizing the importance of repeat ultrasonographic assessment during the third trimester before definitive obstetric management is planned [12,13].

Previous cesarean delivery emerged as the strongest predictor of persistent placental abnormality in the present study, with the likelihood of persistence increasing as the number of previous cesarean deliveries increased. Uterine scarring following cesarean delivery is believed to impair normal decidualization and predispose to abnormal implantation within the lower uterine segment. Multigravidity was also significantly associated with placental persistence, whereas maternal age and previous abortion were not. Although previous uterine instrumentation has been reported as a potential risk factor for abnormal placentation, the available evidence remains inconsistent, and no significant association was observed in the present study [6,14,15].

Women with persistent placental abnormality accounted for all cases of PAS in the present study. In addition, the incidence of cesarean hysterectomy increased with increasing numbers of previous cesarean deliveries, reflecting the cumulative effect of repeated uterine surgery on abnormal placental invasion. These findings are consistent with previous studies demonstrating that placenta previa in the presence of a uterine scar markedly increases the risk of PAS and severe maternal morbidity, underscoring the importance of early identification and planned multidisciplinary management of these pregnancies [5,6,16-18].

Ultrasonography remains the primary modality for antenatal diagnosis of PAS because of its high diagnostic accuracy and ability to identify characteristic imaging features before delivery. Current International Federation of Gynecology and Obstetrics (FIGO) recommendations advocate systematic ultrasonographic evaluation of women with placenta previa or low-lying placenta, particularly in those with previous cesarean delivery, to facilitate early diagnosis and referral to specialized centers [5,6]. Despite excellent diagnostic performance in experienced hands, ultrasonography is influenced by operator expertise, placental location, maternal body habitus, and gestational age, prompting continued interest in adjunctive biomarkers that may improve risk stratification.

Consistent with previous reports, elevated MS-AFP levels were significantly associated with persistent placental abnormality and PAS in the present study. Several investigators have reported higher maternal serum alpha-fetoprotein concentrations among women with placenta accreta spectrum or placental adherence, suggesting that unexplained MS-AFP elevation may serve as an indirect marker of abnormal placentation [7-11,19-23].

The biological basis for elevated MS-AFP in PAS is likely multifactorial. Defective decidualization, absence or deficiency of the Nitabuch layer, excessive trophoblastic invasion, and disruption of the maternal-fetal interface may facilitate increased transfer of fetal alpha-fetoprotein into the maternal circulation. Placental ischemia and abnormal vascular remodeling may also contribute to elevated maternal serum alpha-fetoprotein concentrations, providing a plausible explanation for the observed association between elevated MS-AFP levels and abnormal placentation [6,7].

Although previous studies have established an association between elevated maternal serum alpha-fetoprotein and abnormal placentation, few have assessed its diagnostic performance specifically in women with second-trimester low-lying placenta. In the present study, ROC analysis identified an MS-AFP threshold of 1.10 MoM as the optimal cut-off for predicting persistent placental abnormality, with an area under the curve of 0.892, sensitivity of 88.9%, specificity of 85.5%, positive predictive value of 83.3%, and negative predictive value of 90.4%. Women with MS-AFP values ≥1.10 MoM also demonstrated significantly increased odds and relative risk of persistent placental abnormality. Unlike previous studies that primarily reported an association between elevated MS-AFP and PAS, the present study provides clinically relevant diagnostic performance measures that may assist in identifying women at increased risk. Nevertheless, the proposed cut-off was derived from a single-center cohort and should be validated in larger multicenter prospective studies before routine clinical application.

The findings of the present study suggest that MS-AFP may be considered an adjunct to, rather than a replacement for, ultrasonography in women with low-lying placenta. Incorporation of MS-AFP into antenatal risk assessment may help identify women who require closer imaging surveillance or referral to tertiary care centers. However, because the present study did not directly compare ultrasonography alone with combined ultrasonography and MS-AFP assessment, the incremental diagnostic benefit of such an approach remains uncertain and warrants further investigation.

The strengths of this study include its prospective design, standardized follow-up until delivery, and comprehensive assessment of placental migration, PAS, and maternal outcomes. In addition to conventional statistical analyses, diagnostic performance was evaluated using ROC analysis, and clinically meaningful effect measures, including odds ratios and relative risks, were calculated. Furthermore, the study proposes a clinically interpretable MS-AFP threshold that may provide a foundation for future validation studies.

This study has several limitations. It was conducted at a single tertiary care center with a relatively small sample size, which may limit the generalizability of the findings. Histopathological confirmation of PAS was available only for women who underwent cesarean hysterectomy, whereas women managed conservatively could not be uniformly confirmed pathologically. Multivariable regression analysis was not performed because of the limited number of outcome events, and residual confounding cannot be excluded. Additionally, the proposed MS-AFP threshold has not undergone external validation and should therefore be considered exploratory until confirmed in larger multicenter prospective studies.

In conclusion, elevated maternal serum alpha-fetoprotein measured during the second trimester was strongly associated with persistent placental abnormality and placenta accreta spectrum among women with low-lying placenta. An MS-AFP threshold of 1.10 MoM demonstrated good diagnostic performance for predicting persistent placental abnormality and may serve as a useful adjunct to ultrasonography for antenatal risk stratification. Further prospective multicenter studies are required to validate these findings and determine the clinical utility of incorporating maternal serum alpha-fetoprotein into existing screening strategies.

## Conclusions

Elevated maternal serum alpha-fetoprotein measured during the second trimester is significantly associated with persistence of low-lying placenta and demonstrates good diagnostic performance for identifying women at an increased risk of persistent placental abnormality. When interpreted alongside ultrasonographic findings and established clinical risk factors, MS-AFP may improve antenatal risk stratification and facilitate timely referral for multidisciplinary management. Further multicenter studies are required to validate the proposed cut-off value and determine its applicability in routine obstetric practice.
